# Elimination or Resurgence: Modelling Lymphatic Filariasis After Reaching the 1% Microfilaremia Prevalence Threshold

**DOI:** 10.1093/infdis/jiz647

**Published:** 2019-12-19

**Authors:** Joaquin M Prada, Emma L Davis, Panayiota Touloupou, Wilma A Stolk, Periklis Kontoroupis, Morgan E Smith, Swarnali Sharma, Edwin Michael, Sake J de Vlas, T Déirdre Hollingsworth

**Affiliations:** 1 School of Veterinary Medicine, Faculty of Health and Medical Sciences, University of Surrey, Guildford, UK; 2 Zeeman Institute for Systems Biology and Infectious Disease Epidemiology Research, Mathematics Institute and School of Life Sciences, University of Warwick, Coventry, UK; 3 Department of Statistics, University of Warwick, Coventry, UK; 4 Department of Public Health, Erasmus University Medical Center, Rotterdam, The Netherlands; 5 Department of Biological Sciences, University of Notre Dame, South Bend, Indiana, USA; 6 Big Data Institute, Li Ka Shing Centre for Health Information and Discovery, Headington, Oxford, UK

**Keywords:** lymphatic filariasis, resurgence, elimination, modelling, breakpoints, thresholds, postvalidation surveillance

## Abstract

The low prevalence levels associated with lymphatic filariasis elimination pose a challenge for effective disease surveillance. As more countries achieve the World Health Organization criteria for halting mass treatment and move on to surveillance, there is increasing reliance on the utility of transmission assessment surveys (TAS) to measure success. However, the long-term disease outcomes after passing TAS are largely untested. Using 3 well-established mathematical models, we show that low-level prevalence can be maintained for a long period after halting mass treatment and that true elimination (0% prevalence) is usually slow to achieve. The risk of resurgence after achieving current targets is low and is hard to predict using just current prevalence. Although resurgence is often quick (<5 years), it can still occur outside of the currently recommended postintervention surveillance period of 4–6 years. Our results highlight the need for ongoing and enhanced postintervention monitoring, beyond the scope of TAS, to ensure sustained success.

Elimination of lymphatic filariasis (LF), a filarial nematode infection that falls under the umbrella of neglected tropical diseases, has been on the global agenda since publication of a 1993 report by the International Task Force for Disease Eradication that identified it as 1 of 6 human diseases deemed potentially eradicable with current tools [1]. This led to the adoption of World Health Organization (WHO) resolution 50.29 in 1997, calling for the elimination of LF as a public health problem, with mass drug administration (MDA) as the main strategy. WHO launched the Global Programme to Eliminate Lymphatic Filariasis in 2000 and the resulting global initiative has seen unprecedented international scale up [2].

Elimination as a public health problem (EPHP) is operationalized for LF by the WHO as passing a series of transmission assessment surveys (TAS) that were initially designed to test for a microfilariae (mf) prevalence of less than 1% in areas where *Anopheles* or *Culex* are the main vector; where *Aedes* is the main vector this is 0.5% [3]. As new diagnostics have become available, the current measure used is an antigenemia prevalence of 2%, as a conservative proxy for the historical 1% mf prevalence. Current MDA guidelines advise a minimum of 5 rounds of treatment before a pre-TAS is used to determine whether a first full TAS should be conducted, known as TAS-1. MDA can be stopped if TAS-1 is passed. Two subsequent surveys must also be passed before EPHP can be validated, TAS-2 and TAS-3, each within 2–3 years of the previous assessment.

Reaching <1% mf prevalence was expected to naturally lead to elimination of transmission, following the example of epidemiological studies in China from the 1990s [4]. As of 2017, 11 of the 73 countries listed by WHO as endemic for LF have been validated for EPHP, with 10 more under postintervention surveillance and 46 currently delivering ongoing MDA [5]. The required treatment duration can be considerably longer than the initially anticipated 5–6 years, possibly due to unfavorable transmission or suboptimal program performance [6]. Ten countries have yet to begin MDA [2, 6].

However, recent evidence suggests that the TAS is not capable of detecting ongoing transmission; an example is the low-level persistence in some regions of Sri Lanka despite passing TAS and validation of EPHP in 2016 [7]. These findings prompt concerns that halting interventions could result in low-level maintenance of infection, potentially leading to future resurgence.

Mathematical models can help to assess which conditions are most likely to lead to resurgence and estimate the likelihood of permanently halting transmission, by simulating different scenarios across a range of settings [8–13]. Resurgence is generally considered to refer to a return to baseline levels of infection endemicity following the cessation of MDA.

There is well-supported mathematical and biological theory suggesting a transmission breakpoint for helminth infections, such as LF, which rely on sexual reproduction inside the host [14]. Both male and female parasites are required for reproduction to occur, so infections with a low worm burden are less likely to be infectious. As overall prevalence is pushed down, the mean worm burden also decreases, leading to less onward transmission; eventually sustained transmission becomes nonviable and the disease is expected to naturally die out. This is called the transmission breakpoint and is likely to depend on local transmission conditions, such as biting rate and exposure heterogeneity, as well as biological disease characteristics, such as the extrinsic inoculation period duration (ie, the time required for the parasite to develop in the vector) [15, 16]. Previous studies have suggested that the breakpoint may be substantially below 1% mf prevalence in a number of settings [17, 18]. Stochastic extinction can still occur above this breakpoint but with lower probability [19]. If interventions are halted soon after reaching the breakpoint, low-level residual transmission will decline slowly and it can take a long time for the parasite population to go extinct.

For the current study, multiple modelling groups joined forces to investigate the determinants and timelines of resurgence and elimination, as well as the suitability of a 1% mf threshold. Three well-established transmission models were used to provide greater robustness to our predictions. We did not explicitly model the TAS, instead we focused on the implications of a 1% mf threshold, presenting results that could inform the present global situation and any future developments in transmission assessment methods. As an mf prevalence of <1% in sentinel and spot-check sites is used as the main criteria for implementing TAS [3], our results should be representative of post-MDA settings, although care should be taken when generalizing conclusions to assess TAS results, which currently use an antigenemia prevalence of 2% as a threshold.

## METHODS

Through using models to simulate population and individual trends in infection over time, we investigated a range of scenarios that start the MDA program in 2014 (baseline), do 5 annual rounds, and examine the post-MDA period (2019–2029) for all simulations that achieve the 1% mf threshold 1 year after the fifth round of treatment. We first stratified simulations into 3 categories: resurgence, true elimination, and low-level maintenance of infection (full definitions below). We then used the proportion of simulations in each category as estimates for outcome probabilities and used simulation trajectories to investigate timelines. We also tested a range of potential post-MDA markers of resurgence and elimination that could be used, the best of which are presented here.

### Employed Mathematical Models

We used 3 published models of LF transmission: EPIFIL [9, 20], LYMFASIM [21, 22], and TRANSFIL [16, 23]. EPIFIL is a population-based deterministic model, while both LYMFASIM and TRANSFIL are stochastic individual-based models. All 3 models are age structured; EPIFIL and TRANSFIL are implemented with a balanced birth-death process. A small importation rate is used to allow maintenance of low-level prevalence at equilibrium. The models capture the basic processes relevant to transmission of LF, such as vector density and biting rate, parasite lifecycle, and human exposure to the vectors. The formulation and parametrization of these models has been discussed previously [12] (see also Supplementary Material).

All models can be used to simulate the impact of annual MDA, considering the target coverage, systematic nonadherence patterns (only in LYMFASIM and TRANSFIL, with slightly different, but equivalent, formulations [24]) and the efficacy of employed drugs.

### Scenario Settings

Our simulations here are focused on areas with bancroftian filariasis and anopheles as the dominant vector species. We constrained the baseline mf prevalence before intervention to a range of 5% to 15%, restricting our analysis to the simulations that achieved the 1% mf threshold within the WHO-recommended 5 rounds of annual MDA, with the treatment regime most common in the majority of African settings where *Anopheles* is the vector, which is a combination of ivermectin and albendazole. We provide additional simulation results for regions without onchocerciasis that use a treatment of diethylcarbamazine and albendazole [10, 25], as well as with *Culex* mosquitoes as the dominant vector (Supplementary Material). Individuals aged 5 years and above were used to calculate mf prevalence. We excluded higher-prevalence areas because they usually do not reach the 1% mf threshold after 5 rounds. We also assumed a constant 34% bed net coverage throughout, with this coverage maintained post-MDA until the end of the simulations in 2029.

We considered a broad range of population sizes, with each value drawn from a distribution representative of rural African communities. We assumed most populations are small (median = approximately 1500 individuals), but a handful of locations are highly populated (maximum = 12 000 individuals) (Supplementary Material). We assumed homogeneous mixing in these populations.

In each model, there were a number of parameters that we expected to vary across different populations and settings. These parameters were generally related to transmission, such as vector-host ratio or vector density (see Supplementary Material for further details). For our simulations, we drew values for these parameters from broad prior distributions, based on previous applications of these models [12].

### Forward Simulations

We ran 100 000 simulations for each model. The models were run to endemic equilibrium before MDA, giving the baseline for 2014, and we filtered simulations to generate a uniform distribution in the range between 5% and 15% baseline mf prevalence. We then simulated annual MDA treatment, with a 65% coverage for 5 years. We assumed that a single dose of ivermectin and albendazole kills 35% of adult worms, with 9 months of sterilization for surviving worms, and kills 99% of mf [12]. We selected the simulations that achieved the 1% mf threshold by 2019: about 80% for EPIFIL, 11% for LYMFASIM, and 9% for TRANSFIL of all the runs in the baseline prevalence range in 2014 considered. It is important to note that we selected the simulations based on the true mf prevalence value in the population at 2019 and there was no sampling protocol simulated (ie, TAS). Selected simulations were then run for another 10 years, until 2029, to assess resurgence.

### Analysis of Simulation Results

We defined resurgence 10 years post-MDA as any single return to a greater than 1% mf prevalence during the last 5 years of this period (in this case, 2025–2029); simulations that fulfilled this criterion were classified as resurgent scenarios. For the stochastic models, we can define true elimination as any instance where mf prevalence hits true zero in a population before 2029; in the deterministic model we defined theoretical elimination as an mf prevalence of below 0.1% by 2029. Anything in between resurgence and elimination was considered to be low-level maintenance which, due to long timelines to elimination, may still eventually reach zero prevalence.

Based on these definitions, we could identify which runs lead to resurgence and which do not. We used bootstrap to estimate a 95% confidence interval (CI) for the probability of resurgence. Moreover, we could calculate how many simulations achieved true elimination by 2029. The structural differences between the models give different information. EPIFIL is deterministic and thus we can use it to assess the likely threshold below which resurgence does not occur. The model is run in a Bayesian framework accounting for parameter uncertainty. The lowest mf prevalence value in this model that yields a resurgent simulation was considered an estimate of the theoretical prevalence breakpoint for disease transmission [18, 26]. On the other hand, LYMFASIM and TRANSFIL are stochastic, and thus can inform the probability of resurgence and when it is likely to occur. The timing of resurgence was estimated by pooling the simulations from the 2 stochastic models and finding, for the resurgent simulations, the first year (after 2019) that mf prevalence rose above the threshold.

We then explored options of early warning signs for resurgence, that is are there particular trends that can inform whether resurgence is likely to occur? We looked at 2 possible relevant metrics that could be used to detect resurgence [27]: (1) prevalence 1 year after the last round of MDA, which in the model is true mf prevalence but would operationally be measured as antigenemia prevalence in TAS-1; and (2) difference in prevalence between measurements 1 and 3 years following the last MDA round (measures 2 years apart), which represents the difference in true mf prevalence in the time window between TAS-1 and TAS-2. It is important to note that the TAS is not designed to measure a difference in true prevalence in the population. For these metrics, we examined a range of thresholds that could be used to identify resurgence. For the first we explored a threshold between 0% and 1% mf prevalence; if above the threshold, simulations would be classified as resurgence. For the latter we looked at a difference in mf prevalence from −1% to 1%; a negative difference means an increase in prevalence, below the threshold a run would be classified as resurgence. We then plotted the receiver operating characteristic curve (ROC curve) for the range of possible thresholds considered for these 2 metrics (Supplementary Material).

## RESULTS

**Figure 1.**
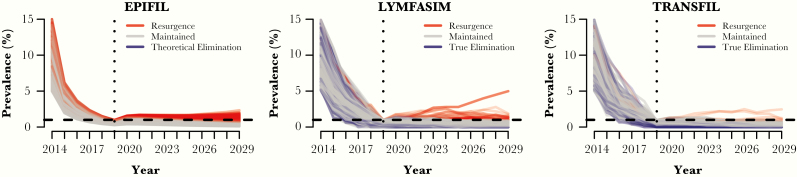
Temporal trends in true microfilaria (mf) prevalence in the population for each individual run. In red are runs that are classified as resurgence, blue are runs that achieve true elimination in LYMFASIM and TRANSFIL (0% mf prevalence) or theoretical elimination in EPIFIL (≤0.1% mf prevalence),, and in grey are runs that by 2029 remain below the threshold but have not achieved true/theoretical elimination.

Figure 1 shows the model-predicted temporal trends in true mf prevalence, during and after 5 years of MDA with albendazole. Based on the lowest mf prevalence value in this model that yielded a resurgent simulation in EPIFIL, the theoretical prevalence breakpoint for disease transmission by this model was estimated at about 0.5% mf prevalence. Resurgence occurred in 1.81% (95% CI, 1.37%–2.4%) of LYMFASIM runs and 0.46% (95% CI, 0.18%–0.89%) of TRANSFIL runs (Figure 1, red), while true elimination (0% mf prevalence by 2029; Figure 1, blue) was achieved in 24% of LYMFASIM runs and 16% of TRANSFIL runs. Conversely, approximately 75% of the LYMFASIM runs and 84% of the TRANSFIL runs saw maintained prevalence below the EPHP threshold without achieving true elimination 10 years later. None of the EPIFIL runs reached the defined theoretical elimination threshold of <0.1% mf prevalence by 2029.

**Figure 2.**
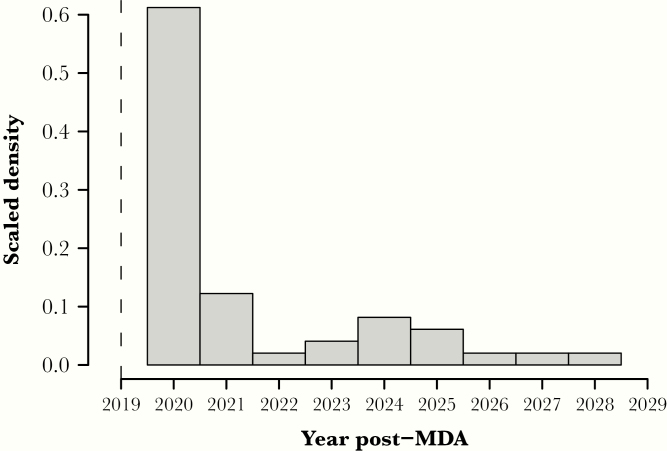
Scaled density histogram of the year of resurgence, defined as the earliest year above the 1% microfilaria (mf) threshold. Simulations from the 2 stochastic models were chosen for mf prevalence of <1% 1 year after mass drug administration (MDA), in 2019, indicated by the dashed line, which represents the timing of transmission assessment survey 1.

We only kept simulations below the 1% mf threshold in 2019. When resurgence occurred it was fairly quick (Figure 2), with 75% (37/49) of the resurgent scenarios in the 2 stochastic models having an mf prevalence returning above the threshold in the first 5 years. However, resurgence may still occur as many as 10 years post-MDA, in the year 2029 itself.

**Figure 3.**
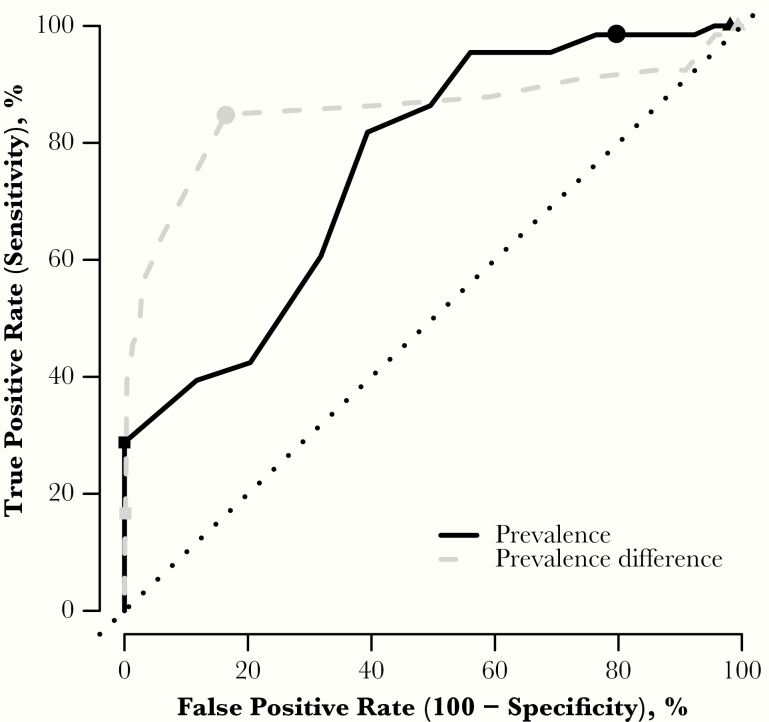
Receiver operating characteristic (ROC) curve showing the true-positive rate against false-positive rate of resurgence for a range of thresholds in 2 different metrics. Black line represents prevalence after mass drug administration (MDA), with a threshold ranging between 0% and 1%. Grey broken line represents the difference in prevalence 1 year after MDA and 2 years later; the threshold ranges from −1% to 1% difference in prevalence. Circles show the 0.5% and zero difference threshold respectively, squares are the 1% prevalence and 0.5% prevalence difference thresholds, and triangles are the 0% and −0.5%, respectively.

To understand the predictability of resurgence, we investigated the sensitivity and specificity of different metrics related to prevalence with possible thresholds (Figure 3, black line). The first metric considered was prevalence a year after MDA. In 98% of runs with resurgence, the mf prevalence 1 year post-MDA was above the EPIFIL-estimated threshold of 0.5% (true-positive rate). However, mf prevalence was also above 0.5% in 73% of runs not resulting in resurgence (false-positive rate). In other words, using a 0.5% threshold we can correctly classify most resurgent simulations; however, many nonresurgent simulations would be also classified as resurgence.

The second metric we considered was the difference in mf prevalence measured 1 and 3 years after the last MDA round (Figure 3, grey line). A negative value represents an increase in prevalence. An intuitive threshold of zero difference (no change) between the 2 years gives a high true-positive rate (approximately 84%) and a relatively low false-positive rate (17%). This cutoff would correctly classify simulations below the threshold (negative difference) as resurgence, while keeping a relatively small proportion of false alarms.

## Discussion

We have used 3 well-established models of LF transmission and control to investigate the potential dynamics of resurgence and elimination for low endemicity settings. In particular, we have focused on the likelihood and timeline of resurgence in scenarios representative of settings with Wuchereria *bancrofti* infections, with *Anopheles* mosquitoes as the dominant vector, and treatment regimens of ivermectin and albendazole.

Our simulations suggest that in situations such as the scenario considered here, with a range of baseline mf prevalence between 5% and 15% before MDA, 5 rounds of annual albendazole campaigns, achieving a 65% coverage and maintaining low-level vector control, resurgence is possible in populations reaching the 1% mf prevalence public health threshold. The likelihood of resurgence is found to be very rare in the 2 stochastic models, TRANSFIL and LYMFASIM, while the deterministic model, EPIFIL, suggests a theoretical transmission breakpoint below the EPHP threshold of 1% mf prevalence.

Obviously, resurgence will be more common in smaller populations (ie, below 1000 individuals); the 1% threshold means less than 10 people infected, and thus a few additional infections can lead to extreme prevalence fluctuations. However, there are a few large populations in which we also observe resurgence, and in those cases it represents a coincidentally large number of new infections after 5 rounds of MDA and reaching the EPHP threshold.

When resurgence occurs, it is most likely to be apparent within a few years of stopping MDA (75% in the first 5 years; Figure 2). A 2-year post-MDA difference in mf prevalence is shown to be a potentially useful indicator for anticipating resurgence. The current monitoring strategies recommend a follow-up survey, TAS-2 conducted 2–3 years after TAS-1, and a third survey, TAS-3, after a similar time period following TAS-2 [3]. However, the current TAS framework is not designed to detect a difference in prevalence, but if additional survey methods could be developed to support this, then they could fit sensibly within present TAS timeframes. Our results suggest such a survey structure could allow detection of resurgence in around 75% of cases but that the timescales of EPHP validation could result in up to 25% of resurgence events being missed (see Figure 2). Metrics such as the estimated mf prevalence and difference since the previous survey could be adapted to other diagnostics (ie, antigenemia) and help inform the likelihood of such resurgence events.

Aiming to test for mf prevalences of less than 1% would present difficulties with survey affordability and sample size but would allow for greater confidence in program success. However, if it was possible to measure 0.5% mf prevalence then this could be sufficient, in scenarios such as those investigated here, to be confident that resurgence is very unlikely to occur.

True elimination is a slow process and in the scenarios we considered, with a range of population sizes and prevalence values, many runs, although not resurging, are maintained below the threshold for over 10 years without reaching zero prevalence (up to 2029, the considered time horizon), even with continued vector control (assumed 34%). While true elimination is not achieved, there is always a small risk of resurgence, therefore careful monitoring of trends is key. Moreover, although the small importation rate used in the simulations did not play a role in resurgence here, highly endemic neighboring areas could have stronger effects.

We have used multiple models, all of which have been previously validated against data, which allows us to capture a range of dynamics and provides robustness to our predictions. Our choice of definition for resurgence is conservative, including all scenarios where mf prevalence goes above 1% at any time across the 5-year period between 2025 and 2029. Because a large number of simulations have low population sizes, some of the detected resurgence events could represent a handful of additional cases due to stochastic fluctuations rather than a real increasing trend. This may lead to an overestimation of resurgence, but a conservative approach ensures we consider all potentially resurging simulations in our analyses.

As we have considered a limited range of preintervention prevalences and restricted ourselves to scenarios that achieve the 1% mf threshold within the minimum recommended 5 rounds of MDA, we would expect our results to provide a best-case scenario of resurgence. As such, resurgence is likely to occur with higher frequency across highly endemic settings, although we would also expect this to be faster and hence potentially more easily detectable within the TAS framework. Qualitatively similar results, with some minor differences, were obtained for additional simulations in settings with an albendazole drug combination and with *Culex*, rather than *Anopheles*, as the dominant vector (shown in the Supplementary Material).

Our results are based on an assumed knowledge of true mf prevalence and we do not directly model antigen tests or the TAS sampling process. In addition, our community-level simulations of prevalence are not necessarily directly comparable to the TAS use of school-based assessment across villages in a larger area.

Our work also highlights that it is not easy to predict resurgence or elimination based on current prevalence only, due to the lack of specificity; metrics such as prevalence difference are seen to be better indicators (ROC curve; Figure 3). This implies that ongoing monitoring is still required beyond TAS timelines to ensure reductions in disease are maintained and detect whether a break in transmission has been achieved.

In summary, resurgence is generally rare with fairly conservative assumptions; however, many instances occur outside of the TAS1–3 period, therefore careful monitoring, even 5 years after cessation of MDA, would be recommended. Achieving true elimination is a slow process, and even in a 10-year time horizon, many runs do not reach it. Our analysis is based on true mf prevalence and therefore reliable diagnostics are necessary to detect possible resurgence rapidly.

## Supplementary Data

Supplementary materials are available at *The Journal of Infectious Diseases* online. Consisting of data provided by the authors to benefit the reader, the posted materials are not copyedited and are the sole responsibility of the authors, so questions or comments should be addressed to the corresponding author.

jiz647_suppl_Supplementary_Material

## Notes

***Author contributions*.** J. M. P., E. L. D., P. T., and T. D. H. developed the study conception and design. Model simulations were performed by M. S. and S. S. (EPIFIL), W. A. S. and P. K. (LYMFASIM), and P. T. (TRANSFIL). J. M. P. carried out analyses. J. M. P., E. L. D., and W. A. S. drafted the manuscript. All authors helped in the interpretation of results and critically reviewed the manuscript.

***Acknowledgment.*** We kindly thank Dr Johanna Muñoz for helping with additional runs of LYMFASIM during the review process.

***Disclaimer***. The funders had no role in study design, data collection and analysis, decision to publish, or preparation of the manuscript.

***Financial support.*** This work was supported by the Bill and Melinda Gates Foundation through the NTD Modelling Consortium (grant number OPP1184344).

***Supplement sponsorship.*** The authors acknowledge funding of the NTD Modelling Consortium by the Bill and Melinda Gates Foundation (OPP1184344).

***Potential conflicts of interest.*** All authors: No reported conflicts of interest. All authors have submitted the ICMJE Form for Disclosure of Potential Conflicts of Interest. Conflicts that the editors consider relevant to the content of the manuscript have been disclosed.

Presented in part: Annual Neglected Tropical Diseases Modelling Consortium Spring Technical Meeting, University of Oxford, Oxford, UK, 18–20 March 2019.
