# Supplementary material for: Elimination or Resurgence: Modelling Lymphatic Filariasis After Reaching the 1% Microfilaremia Prevalence Threshold
Source: J Infect Dis. 2019 Dec 19;221(Suppl 5):S503–9. doi: 10.1093/infdis/jiz647 (PMC7289550; doi:10.1093/infdis/jiz647)
Supplement: jiz647_suppl_Supplementary_Material [file jiz647_suppl_supplementary_material.pdf]

Supplementary Material

Prada, Davis *et al.* 2019

Index

Population size distribution	2
Receiver Operating Characteristic (ROC) curve	3
EPIFIL model description and methods	4
LYMFASIM model description and methods	12
TRANSFIL model description and methods	26
Exploring alternative scenarios	31

Population size distribution

The models considered a range of population sizes, with a distribution broadly representative of rural communities in lymphatic filariasis endemic regions. The distribution used can be seen in Figure S1, with most population being fairly small, and a few larger towns. We limited the population size to 12000, as with higher population sizes, the computation time is increased and some of the assumptions in the models (such as homogeneous mixing) are less realistic. We extracted the distribution from the Worldpop database[1], we used the populations of 5x5 km "pixels" (i.e. squares) as our populations sizes, based on the LF endemic region in South-West Ethiopia.

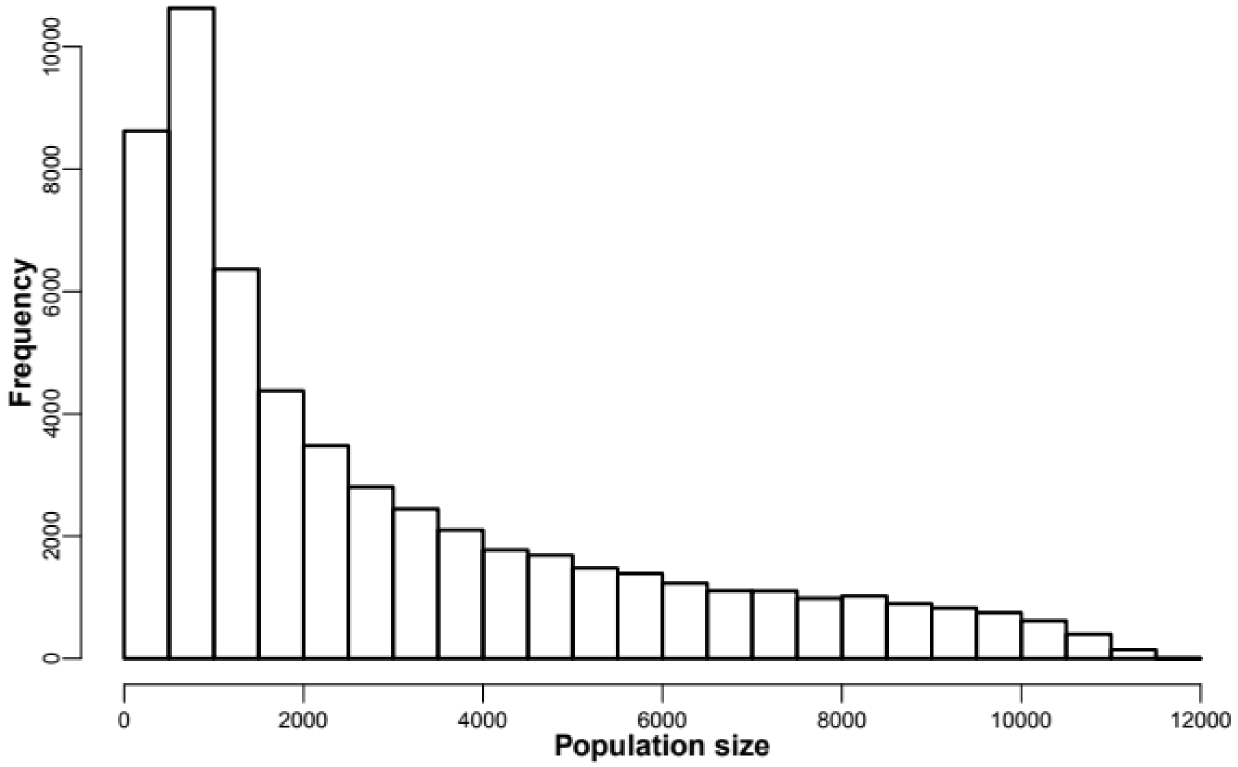

Figure S1 – Histogram of population sizes used, based on the population size of 5x5 km pixels in LF endemic regions.

Reference
1. https://www.worldpop.org/

Receiver Operating Characteristic (ROC) curve

The ROC curve is a graphical illustration that plots the true positive rate (TPR) against the false positive rate (FPR). The true positive rate is also known as the sensitivity, and in our case, it represents the proportion of simulations classified as resurgence that are above a certain threshold from among all simulations that resurge. Conversely, the false positive rate is the proportion of simulations that are not resurging but are above the threshold from among all simulations that are not resurging. The FPR is also equal to 1 – specificity (or 100 – specificity if using the rates as percentages).

In our implementation, we pooled together the simulations from both LYMFASIM and TRANSFIL (the two stochastic models). The first metric considered was the prevalence after 5 rounds of MDA (1 year since the last round), which represents TAS-1. We varied the possible threshold from a mf prevalence of zero to a mf prevalence of 1%. The breakpoint in the deterministic model EPIFIL was at a prevalence of 0.5%; using that threshold yields a TPR of 95%, but a FPR of 70% (which is a specificity of 30%). The second metric considered was the difference in prevalence from the first year after MDA and two years later, this represents measuring the difference in prevalence between TAS-1 and TAS-2. We varied the possible threshold from -1% to 1% in mf prevalence difference. A negative difference means that TAS-2 prevalence is higher than TAS-1, while a positive prevalence means that prevalence decreased from TAS-1 to TAS-2. An intuitive natural threshold to consider is zero difference (i.e. TAS-1 and TAS-2 yield the same mf prevalence), and this threshold has a TPR of 80% and a FPR of 10% (which is a specificity of 90%). We initially considered other similar metrics to Truscott and colleagues[1], however none of those metrics had any discriminatory power in our results.

It is important to note that even a low FPR (such as the 10% obtained with a threshold of zero difference between TAS-1 and TAS-2), would still misclassify many simulations as resurgence (as there are 6-7 resurgent simulations per 1000 runs).

Reference

1. Truscott, J. et al. Identifying optimal threshold statistics for elimination of hookworm using a stochastic simulation model. Parasites & Vectors **2017**; 10.

EPIFIL model description and methods

The mathematical model of LF transmission dynamics

We employed a genus specific mosquito-vectored transmission model of LF to carry out the modelling work in this study [1-7]. Briefly, the state variables of this hybrid coupled partial differential and differential equation model vary over age (*a*) and/or time (*t*), representing changes in the pre-patent worm burden per human host $(P(a,t)),$ adult worm burden per human host $(W(a,t)),$ the microfilariae (Mf) level in the human host modified to reflect infection detection in a 1 mL blood sample $(M(a,t)),$ the average number of infective L3 larval stages per mosquito (*L*), and a measure of immunity $(I(a,t))$ developed by human hosts against L3 larvae. The state equations comprising this model are:

$$\frac{\partial P}{\partial t}+\frac{\partial P}{\partial a}=\lambda\frac{V}{H}h(a)\Omega(a,t)-\mu P(a,t)-\lambda\frac{V}{H}h(a)\Omega(a,t-\tau)\zeta$$

$$\frac{\partial W}{\partial t}+\frac{\partial W}{\partial a}=\lambda\frac{V}{H}h(a)\Omega(a,t-\tau)\zeta-\mu W(a,t)$$

$$\frac{\partial M}{\partial t}+\frac{\partial M}{\partial a}=\alpha s\phi[W(a,t),k]W(a,t)-\gamma M(a,t)$$

$$\frac{\partial I}{\partial t}+\frac{\partial I}{\partial a}=W_T(a,t)-\delta I(a,t)$$

$$\frac{dL}{dt}=\lambda\kappa g\int\pi(a)(1-f[M(a,t)])da-(\sigma+\lambda\psi_1)L$$

$$L^*=\frac{\lambda\kappa g\int\pi(a)(1-f[M(a,t)])da}{\sigma+\lambda\psi_1}$$

The above equations involve partial derivatives of four state variables (*P* - pre-patent worm load; *W* - adult worm load; *M* - microfilaria intensity; *I* - immunity to acquiring new infection due to the pre-existing total worm load where $W_T = W(a,t) + P(a,t)$). Given the faster time scale of infection dynamics in the vector compared to the human host, the infective L3-stage larval density in mosquito population is modelled by an ordinary differential equation essentially reflecting the significantly faster time-scale of the infection dynamics in the vector hosts. This allows us to make the simplifying assumption that the density of infective stage larvae in the vector population reaches a dynamic equilibrium (denoted by L^*) rapidly[1, 2, 5, 8, 9]. This basic coupled immigration-death structure of the model as well as its recent extensions has been extensively

discussed previously[1-3, 5, 8, 9]. The effects of worm patency are captured by considering that at any time *t*, human individuals of age less than or equal to the pre-patency period, *τ*, will have no adult worms or Mf, and the rate at which pre-patent worms survive to become adult worms in these individuals at *a* > *τ* is given by $\zeta = \exp(-\mu\tau)$. The term enables us to account for the different establishment and development rates of the incoming L3-stage larvae as adult worms depending on the genus of mosquito vectors as expressed below:

$$f[M(a,t)] = \left[\frac{2}{\left[1+\frac{M(a,t)}{k}\left(1-\exp\left[-\frac{r}{\kappa}\right]\right)\right]^{k}} - \frac{1}{\left[1+\frac{M(a,t)}{k}\left(1-\exp\left[-\frac{2r}{\kappa}\right]\right)\right]^{k}} \right]$$

for mosquitoes of *Anopheline* genus;

$$f[M(a,t)] = \left(1+\frac{M(a,t)}{k}\left(1-\exp\left[-\frac{r}{\kappa}\right]\right)\right)^{-k}$$

for mosquitoes of *Culicine* genus.

In the above, $k[=k_0 + k_{Lin}M]$ is the shape parameter of the negative binomial distribution on the Mf uptake whereas r and are respectively the rate of initial increase and the maximum level of L3 larvae. See Table 1 for the description of all the model parameters and functions.

Table S1 – Description of EPIFIL model parameters and functions.

Parameter	Definition (*units*)	Range	Refs
λ	Number of bites per mosquito (*per month*)	[5, 15]	1, 2, 5, 10, 11
τ	Pre-patency period	[1, 9]	12
s	Proportion of female worms	0.5	-
μ	The worm mortality rate (*per month*)	[0.008, 0.018]	1, 2, 5, 13-16
α	Production rate of microfilariae per worm (*per month*)	[0.25, 1.5]	1, 2, 5, 17
γ	The death rate of the microfilariae (*per month*)	[0.08, 0.12]	1, 5, 15, 17
g	Proportion of mosquitoes which pick up infection when biting an infected host	[0.251, 0.485]	1, 5, 18
κ	Maximum level of L3 given Mf density	[3, 5]	1, 5
k_0	The basic location parameter of negative binomial distribution used in aggregation parameter ($k = k_0 + k_{Lin} M$)	[0.000036, 0.000775]	1, 5, 19, 20
δ	Immunity waning rate (*per month*)	[0.001, 0.01]	1, 5
V	Vector population size	[25000, 100000]	data
H	Human population size	data	data
k_{Lin}	The linear rate of increase in the aggregation parameter defined above	[0.00000024, 0.282]	1, 5, 19, 20
σ	Death rate of mosquitoes (*per month*)	[1.5, 8.5]	1, 5, 20
ψ_1	Proportion of L3 leaving mosquito per bite	[0.1, 0.8]	17
ψ_2	The establishment rate[1]	[0.00003, 0.00364]	1, 2, 5, 21
H_{Lin}	A threshold value used in *h(a)* to adjust the rate at which individuals of age *a* are bitten: linear rise from 0 at age zero to 1 at age H_{Lin} in years. $h(a) = a / H_{Lin}$ for $a < H_{Lin}$; $h(a) = 1$ for $a \geq H_{Lin}$	[240, 360] months	1, 5, 9
r	Gradient of Mf uptake[2]	[0.04, 0.25]	1, 5
c	Strength of acquired immunity	[0.015, 0.025]	1, 5
I_C	Strength of immunosuppression[3]	[0.5, 5.5]	1, 5
S_C	Slope of immunosuppression function[4] (*per worm/month*)	[0.01, 0.20]	1, 5
	Intervention-related parameters		
ω	Worm killing efficacy of drug (instantaneous)	dependent on drug regimen	3
ε	Microfilariae killing efficacy of drug (instantaneous)	dependent on drug regimen	3
δ_{reduc}	Reduction in the worm's fecundity over a period of time *p* due to drug	dependent on drug regimen	3
p	A time period during which the drug remains efficacious in reducing the fecundity of the surviving adult worms	dependent on drug regimen	3
C	Percentage of the population administered the drug	data	data
MBR_{VC}	Vector control (VC) modifies V/H ($= MBR/\lambda$) where	data and estimates	19, 20

	$MBR_{VC} = MBR_0 \exp[a_1 t]$, with $a_1 < 0$ for $\forall t$ when VC is implemented, otherwise $a_1 > 0$.		
**Description**	**Mathematical expressions of the functions**	**Parameters**	
Probability that an individual is of age *a* ***π(a)***	$\pi(a) = A_0 \exp[-B_0 a]$	Human age *a* in month, A_0 and B_0 estimated from country demographic data	1, 5, 9
Larvae establishment rate (modified by acquired immunity) ***Ω(a,t)***	$L^* \psi_1 \psi_2 g_1(I) g_2(W_T)$	ψ_1 - proportion of L3 leaving mosquito per bite; ψ_2 - the establishment rate[1]	-
Adult worm mating probability ***ϕ(W,k)***	$1 - \left(1 + \frac{W}{2k}\right)^{-(1+k)}$	*k* – negative binomial aggregation parameter	2, 5, 22
Immunity to larval establishment $g_1(I)$	$\frac{1}{1+cI}$	*c* – strength of immunity to larval establishment	1, 5
Host immunosuppression $g_2(W_T)$	$\frac{1 + I_C S_C W_T}{1 + S_C W_T}$	I_C – strength of immunosuppression; S_C – slope of immunosuppression	1, 5

[1]The proportion of L3-stage larvae infecting human hosts that survive to develop into adult worms[2].
[2]The gradient of Mf uptake *r* is a measure of the initial increase in the infective L3 larvae uptake by vector as *M* increases from 0[2, 9].
[3] The facilitated establishment rate of adult worms due to parasite-induced immunosuppression in a heavily infected human host
[4] The initial rate of increase by which the strength of immunosuppression is achieved as *W* increases from 0[23].
Note MBR (monthly biting rate) serves as an input to initialize the model, measured as mosquito bites per person per month, the value of which may be obtained from entomological surveys conducted in study sites. In the absence of the observed MBR value, the model has been adapted to estimate it from the community-level Mf prevalence data.

Model implementation

Sampling population sizes

In this work, we were interested in simulating infection prevalence scenarios for the expected range of village population sizes in rural African communities. Typically, we sample the ratio of vectors to humans as a composite parameter (*V/H*). To explicitly account for the human population size, we instead sampled values for *H*, human population, and *V*, vector population separately. *H* was sampled from the provided population size distributions *V* was sampled from a uniform distribution.

Parameter selection and simulation procedure

We employed the Bayesian Melding (BM) procedure to calibrate and estimate the LF models from field data, as outlined in detail in our previous work [2, 5-7]. Typically, we begin the procedure by first using the known or assignment of a uniform range for parameter values to generate distributions of parameter priors. We then randomly sample with replacement from these prior distributions to generate 200,000 parameter vectors, which are run using the annual biting rate (ABR) values, if given, for a site to generate model outputs. The model outputs are then melded with age-stratified Mf prevalence data by calculating binomial log-likelihoods for each parameter vector. In the resampling step of the BM method, a Sampling-Importance-Resampling (SIR) algorithm is used to perform 500 draws with replacement from the pool of parameter vectors generated as above, with probabilities proportional to their relative log likelihood values. This step selects the parameter vectors which best describe the given mf age-prevalence data. These resampled parameter vectors are then used to generate distributions of variables of interest from the fitted model (eg. age-prevalence curves, worm breakpoints, and infection trajectories following treatments).

Here, we modified our standard Monte-Carlo BM framework for model discovery [2, 5-7, 24] to provide simulations for the chosen scenarios in Ethiopia. The explicit aim was to generate at least N = 100,000 parameter vectors which resulted in a uniform distribution of overall mf prevalence values 5-15% at baseline in 2014. The Anopheline-transmitted LF model was used. We first randomly sampled n = 100,000 parameter vectors from the assigned uniform parameter priors. We then simulated the endemic equilibrium given each parameter vector and calculated the predicted mf prevalence at baseline. Those parameter vectors whose outputs produced mf prevalence values between 5-15% were accepted while all others are rejected. This sampling and acceptance/rejection procedure was repeated until the total number of accepted parameter vectors (N) was greater than or equal to 100,000. To ensure that a uniform posterior distribution of prevalences was generated by this approach, a check was made to confirm that the modeled prevalences fell into uniform bin sizes in prevalence range. The N posterior parameter vectors were then used to simulate the impacts of MDA and vector control interventions.

Modeling intervention by mass drug administration and bednets

Intervention by mass drug administration was modeled based on the assumptions that anti-filarial treatment with a combination drug regimen acts by killing certain fractions of the populations of adult worms and microfilariae instantly after the drug administration. These effects are incorporated into the basic model by calculating the population sizes of worms and microfilariae as follows:

$$\left.\begin{aligned} P(a,t+dt) &= (1-\omega C)P(a,t) \\ W(a,t+dt) &= (1-\omega C)W(a,t) \\ M(a,t+dt) &= (1-\varepsilon C)M(a,t) \end{aligned}\right\} \quad \text{at time } t = T_{MDA_i}$$

where *dt* is a short time period since the *i*th MDA was administered. During this short time interval, a given proportion of adult worms and microfilariae are instantly removed. The parameters ω and ε are drug killing efficacy rates for the two life stages of the parasite while the parameter C represents the MDA coverage. Apart from instantaneous killing of microfilariae, the drug continues to kill the newly reproduced Mf by any surviving adult worms at a rate δ_{reduc} for a period of time, p. We model this effect as follows:

$$\frac{\partial M(a,t)}{\partial t} + \frac{\partial M(a,t)}{\partial a} = (1-\delta_{reduc}C)s\alpha\phi(W(a,t),k)W(a,t) - \gamma M(a,t), \quad \text{for } T_{MDA_i} < t \le T_{MDA_i} + p$$

We simulated LF intervention by running the model with fixed values of ω, ε, δ_{reduc}, and p for MDA coverage levels given by the scenarios. The first MDA round was implemented in the model by affecting the population sizes of worms and microfilariae from the baseline estimates, and then the intervention is simulated forward in time for a number of years, with subsequent MDA rounds implemented annually. We did not consider the effects of importation of infection over time.

In addition to MDA, we also modeled the added effect of long lasting insecticidal nets (LLINs) as described previously [6]. The impact of LLINs with three main actions against mosquito biting was modelled: 1) deterrence from entering the home (efficacy η_1), 2) inhibition of their ability to feed on humans (efficacy η_2), and 3) killing them (efficacy η_3) [25, 26]. To capture these effects, which decay over time as the larvicide efficacy declines exponentially at rate Λ, we adjust the term V/H to be appropriately modified according to the population coverage (C_{VC}):

$$\frac{V}{H}\left(1-\eta_1 \exp(-\Lambda t)\, C_{VC}\right)\left(1-\eta_2 \exp(-\Lambda t)\, C_{VC}\right)\left(1-\eta_3 \exp(-\Lambda t)\, C_{VC}\right)$$

References

1. Gambhir, M. & Michael, E. Complex ecological dynamics and eradicability of the vector borne macroparasitic disease, lymphatic filariasis. *PLoS One* **3**, e2874 (2008).

2. Gambhir, M. *et al*. Geographic and ecologic heterogeneity in elimination thresholds for the major vector-borne helminthic disease, lymphatic filariasis. *BMC biology* **8**, 1 (2010).

3. Michael, E. *et al*. Mathematical modelling and the control of lymphatic filariasis. *The Lancet infectious diseases* **4**, 223-234 (2004).

4. Michael, E., Malecela-Lazaro, M. N., Kabali, C., Snow, L. C. & Kazura, J. W. Mathematical models and lymphatic filariasis control: endpoints and optimal interventions. *Trends Parasitol.* **22**, 226-233 (2006).

5. Singh BK and Bockarie MJ and Gambhir M and Siba PM and Tisch DJ and Kazura J and others. in *Sequential Modelling of the Effects of Mass Drug Treatments on Anopheline-Mediated Lymphatic Filariasis Infection in Papua New Guinea* (PLoS One, 2013).

6. Singh, B. K. & Michael, E. Bayesian calibration of simulation models for supporting management of the elimination of the macroparasitic disease, Lymphatic Filariasis. *Parasit vectors* **8**, 1-26 (2015).

7. Michael, E. & Singh, B. K. Heterogeneous dynamics, robustness/fragility trade-offs, and the eradication of the macroparasitic disease, lymphatic filariasis. *BMC medicine* **14**, 1 (2016).

8. Chan, M. S. *et al*. Epifil: a dynamic model of infection and disease in lymphatic filariasis. *Am. J. Trop. Med. Hyg.* **59**, 606-614 (1998).

9. Norman, R. *et al*. EPIFIL: the development of an age-structured model for describing the transmission dynamics and control of lymphatic filariasis. *Epidemiol. Infect.* **124**, 529-541 (2000).

10. Rajagopalan, P. Population dynamics of culex pipiens fatigans, the filariasis vector, in pondicherry: influence of climate and environment. *Proc Ind Nat Science Acad B* **46**, 745-752 (1980).

11. Subramanian, S., Manoharan, A., Ramaiah, K. D. & Das, P. K. Rates of acquisition and loss of Wuchereria bancrofti infection in Culex quinquefasciatus. *Am. J. Trop. Med. Hyg.* **51**, 244-249 (1994).

12. Scott, A. L. & Nutman, T. Lymphatic-dwelling filariae. *Lymphatic filariasis.*, 5-39 (2000).

13. Vanamail, P., Subramanian, S., Das, P. K., Pani, S. P. & Rajagopalan, P. K. Estimation of fecundic life span of Wuchereria bancrofti from longitudinal study of human infection in an endemic area of Pondicherry (south India). *Indian J. Med. Res.* **91**, 293-297 (1990).

14. Evans, D. B., Gelband, H. & Vlassoff, C. Social and economic factors and the control of lymphatic filariasis: a review. *Acta Trop.* **53**, 1-26 (1993).

15. Ottesen, E. & Ramachandran, C. Lymphatic filariasis infection and disease: control strategies. *Parasitology Today* **11**, 129-130 (1995).

16. Vanamail, P. *et al*. Estimation of the fecund life span of Wuchereria bancrofti in an endemic area. *Trans. R. Soc. Trop. Med. Hyg.* **90**, 119-121 (1996).

17. Hairston, N. G. & de Meillon, B. On the inefficiency of transmission of Wuchereria bancrofti from mosquito to human host. *Bull. World Health Organ.* **38**, 935-941 (1968).

18. Subramanian, S. *et al*. The relationship between microfilarial load in the human host and uptake and development of Wuchereria bancrofti microfilariae by Culex quinquefasciatus: a study under natural conditions. *Parasitology* **116**, 243-255 (1998).

19. Subramanian, S., Pani, S., Das, P. & Rajagopalan, P. Bancroftian filariasis in Pondicherry, south India: 2. Epidemiological evaluation of the effect of vector control. *Epidemiol. Infect.* **103**, 693-702 (1989).

20. Das, P. *et al*. Bancroftian filariasis in Pondicherry, south India–epidemiological impact of recovery of the vector population. *Epidemiol. Infect.* **108**, 483-493 (1992).

21. Ho, B. C. & Ewert, A. Experimental transmission of filarial larvae in relation to feeding behaviour of the mosquito vectors. *Trans. R. Soc. Trop. Med. Hyg.* **61**, 663-666 (1967).

22. May, R. M. Togetherness among schistosomes: its effects on the dynamics of the infection. *Math. Biosci.* **35**, 301-343 (1977).

23. Duerr, H., Dietz, K. & Eichner, M. Determinants of the eradicability of filarial infections: a conceptual approach. *Trends Parasitol.* **21**, 88-96 (2005).

24. Poole, D. & Raftery, A. E. Inference for deterministic simulation models: the Bayesian melding approach. *Journal of the American Statistical Association* **95**, 1244-1255 (2000).

25. Griffin, J. T. *et al*. Reducing Plasmodium falciparum malaria transmission in Africa: a model-based evaluation of intervention strategies. *PLoS Med* **7**, e1000324 (2010).

26. Okumu, F. O. & Moore, S. J. Combining indoor residual spraying and insecticide-treated nets for malaria control in Africa: a review of possible outcomes and an outline of suggestions for the future. *Malaria journal* **10**, 1 (2011).

LYMFASIM model description and methods

Description of the mathematical model

LYMFASIM[1,2] is a stochastic individual-based model for lymphatic filariasis (LF). It is a specific model variant within WORMSIM, a generalized framework for modelling transmission and control of helminth infections in humans[3,4]. LYMFASIM simulates the life histories of individual people and individual worms in a community, and the effects of interventions (e.g. mass drug administration, integrated vector management, bednet use) on transmission and morbidity, while taking into account the human demography and the complexities of helminth transmission. The model has been described elsewhere and has been applied to support decision making on control and elimination of lymphatic filariasis in different settings[1,2,5–13].

Mass drug administration (MDA) is simulated by specifying the exact timing of the treatment rounds (year, month), the efficacy of the applied treatment regimen, the achieved coverage level, and compliance patterns. LYMFASIM assumes that a fraction of people never participates in MDA (e.g. systematic refusal, related to chronic illness). In addition, LYMFASIM allows the relative compliance to vary between age and sex groups; this mechanism captures transient contra-indications for MDA (e.g. exclusion of young children and pregnant women) and other age- and sex-related behavioral factors driving participation in MDA. Lastly, each individual has a personal inclination to participate in MDA, which is considered as a lifelong property. A stochastic process eventually defines for each individual whether they are treated in a given round, depending on the calculated probability.

Parameter quantification and simulation methods for this study

To simulate LF transmission by Anopheles and Culex, we used previously derived model parameterization for Africa[8] and India[2]. Parameter values are listed in Table S2. Assumptions and parameters related to control strategies and treatment efficacy are listed in Table S3. The MDA timing and coverage simulated was 5 annual rounds set to 65% coverage. The monthly biting rate (mbr) and exposure heterogeneity parameter (k) were varied to generate simulations across a wide range of mf prevalences at baseline, measured in the total population (all ages). The density plot Figure S2 illustrates the parameter space areas from the Anopheles-simulations that were in the 5 to 15% mf

prevalence range at baseline and reached the 1% mf threshold after 5 rounds of MDA, almost 14000 runs.

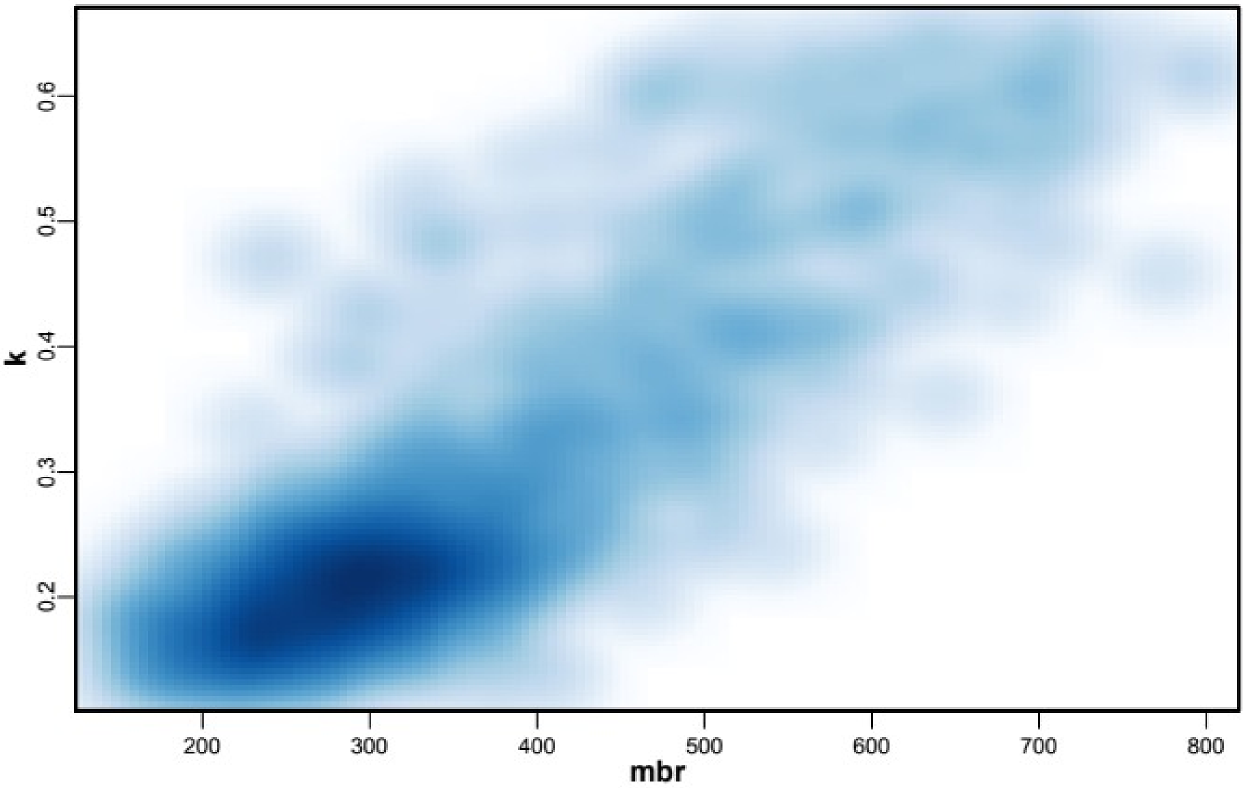

Figure S2 – Monthly biting rate, mbr, against exposure heterogeneity parameter k, for the Anopheles simulations. The density plot illustrates the parameter space areas from the simulations that were in the 5 to 15% mf prevalence range at baseline and reached the 1% mf threshold after 5 rounds of MDA. Dark blue areas denote more common parameter values.

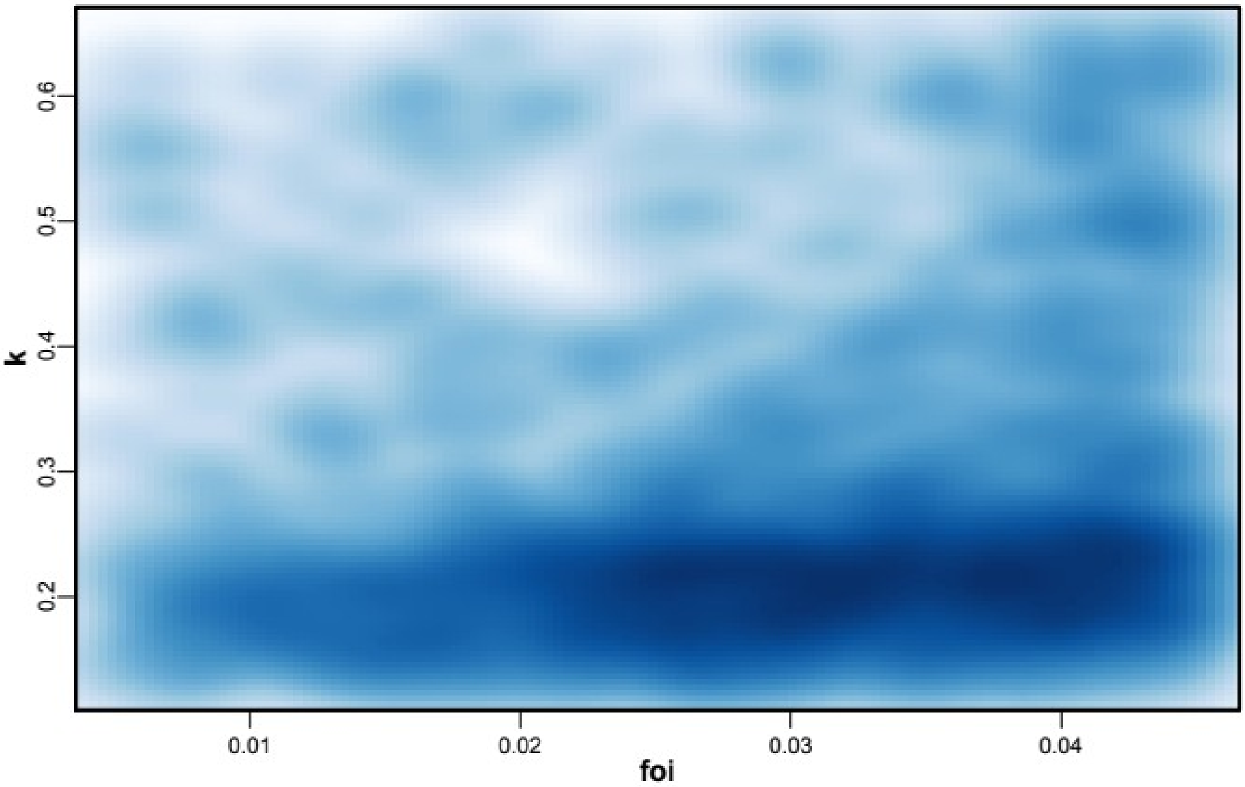

Figure S3 – Force of infection, foi, against exposure heterogeneity parameter k, for the Anopheles simulations. As above, the density plot illustrates the parameter space areas from the simulations that were in the 5 to 15% mf prevalence range at baseline and reached the 1% mf threshold after 5 rounds of MDA. Dark blue areas denote more common parameter values.

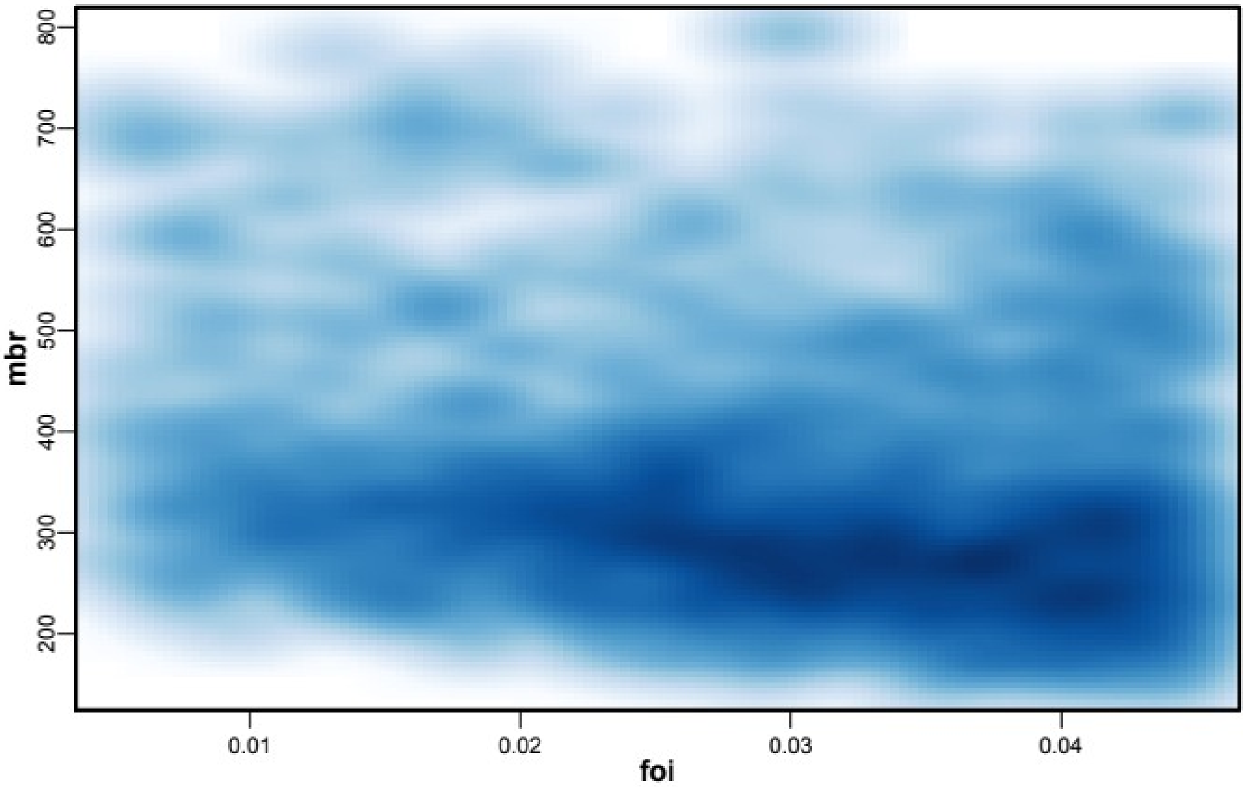

Figure S4 – Force of infection, foi, against monthly biting rate, mbr, for the Anopheles simulations. As above, the density plot illustrates the parameter space areas from the simulations that were in the 5 to 15% mf prevalence range at baseline and reached the 1% mf threshold after 5 rounds of MDA. Dark blue areas denote more common parameter values.

Version and code availability

For this paper, we used WORMSIM version 2.58Ap25. The code for this version of LYMFASIM used in this paper is available from https://gitlab.com/erasmusmc-public-health/wormsim.previous.versions.

Table S2 – LYMFASIM input: probability distributions, functions and parameter values for simulating transmission of bancroftian filariasis by Anopheles mosquitoes in Africa or Culex in India

**Parameter description**	**Anopheles model**		**Culex model**		**Source / remarks**
**Human demography**					
Cumulative survival, by age	Age	Survival	Age	Survival	Anopheles: Modified from ref[8], to match the population composition in Ethiopia; Culex: Fixed as in[2]
	0	1	0	1	
	5	0.800	5	0.904	
	15	0.790	10	0.895	
	20	0.755	15	0.888	
	25	0.737	20	0.879	
	30	0.723	25	0.864	
	35	0.654	30	0.849	
	40	0.605	40	0.812	
	45	0.560	50	0.756	
	50	0.506	90	0	
	60	0.487			
	70	0.305			
	80	0.155			
	99	0.000			
Fertility rate per woman, by age	Age	Fertility rate	Age	Fertility rate	Anopheles: fixed, as in [8]; Culex: fixed as in [2]
	0	0	0	0	
	5	0	5	0	
	15	0	10	0	
	20	0.116	15	0	
	25	0.230	20	0.075	
	30	0.245	25	0.254	
	35	0.207	30	0.222	
	40	0.147	40	0.096	
	45	0.077	50	0.013	
	50	0.031	90	0	
	60	0			

Parameter description	Anopheles model		Culex model		Source / remarks
	70	0			
	80	0			
	99	0			
Initial population	age	Male/females	Age	Males/females	N/A
	5	42/42	5	33/33	
	15	63/63	10	28/28	
	20	26/26	15	25/25	
	25	22/22	20	25/25	
	30	20/20	25	37/37	
	35	17/17	30	33/33	
	40	14/14	40	25/25	
	45	11/11	50	22/22	
	50	9/9	90	22/22	
	60	14/14			
	70	9/9			
	80	3/3			
	99	1/1			
Maximum population size	Varied according to population size distribution defined elsewhere in this manuscript		Varied according to population size distribution defined elsewhere in this manuscript		Figure S1
Proportion removed when maximum population size is reached	5%		5%		N/A
**<u>Exposure</u>**					
External force-of-infection at start of burn-in period	2		2		N/A
Duration of external force-of-infection at start of burn-in period	2 years		2 years		N/A
External force-of-infection (foi) during	Sampled from a uniform distribution		Sampled from a uniform distribution		N/A

Parameter description	Anopheles model	Culex model	Source / remarks
burn-in period	between 0.005 and 0.045; assumed to decline gradually after the introduction of mass treatment in the simulated population	between 0.005 and 0.035; assumed to decline gradually after the introduction of mass treatment in the simulated population	
Average mosquito biting rate for adult men (mbr), for a relative biting rate of 1	Varied between runs, as specified in figure S2	Varied between runs, with values ranging from 100-3000	N/A
Seasonal variation in biting rate	No	No	N/A
Variation in exposure by age (no difference assumed between sexes)	0 at birth, linearly increasing to reach 1 at the age of 20 and constant at 1 from this age onwards	0.26 at birth, linearly increasing to reach 1 at the age of 19.1 and constant at 1 from this age onwards	Anopheles: slightly adjusted from [2] for Africa[8]; Culex: estimated by fitting to data[2];
Probability distribution describing variation in the individual exposure index, due to personal factors (fixed through life) given age and sex	Gamma distribution with mean 1.0 and shape (=rate) varied uniformly with the range [0.1-1] (see Figure S2)	Gamma distribution with mean 1.0 and shape (=rate) varied with the range [0.1-1.4]	Gamma distribution is assumed as in[2,8]; shape/rate parameter varied
**<u>Parasite dynamics within host</u>**			
Success ratio	0.00088	0.00103	Previously estimated by fitting to data[8,2]
Anti-L3 immunity	Not included in the model, by assuming that the strength and duration of the immunological memory are zero	Included in the model assuming the following parameters: Shape-parameter for the gamma-distribution describing individual variation in the ability to develop an anti-L3 immune-response = 1.07	Anopheles: assumed not to play a role[8]; Culex: parameters for

Parameter description	Anopheles model	Culex model	Source / remarks
		Strength of immunological memory for anti-L3 immunity = 0.0000589; duration of immunological memory for anti-L3 immunity, in years = 9.6 years	anti-L3 immunity model were estimated by fitting to to data[2]
Anti-fecundity immunity:	Not included in the model, by assuming that the strength and duration of the immunological memory are zero	Not included in the model, by assuming that the strength and duration of the immunological memory are zero	Assumed[8,2]
Average worm lifespan	10 years on average; varied according to a Weibull distribution with shape 2	10.2 on average; varied according to a Weibull distribution with shape 2	Previously estimated by fitting to data[2]
Duration of immature stage of the parasite in human host	Constant, 8 months	Constant, 8 months	Fixed, based on [14]
No. of Mf produced per female parasite per month per 20 ml peripheral blood in the absence of immune reactions and in the presence of at least 1 male worm	0.58	0.606	Previously estimated by fitting to data[2]
Monthly survival of the microfilariae, fraction	0.9	0.9	Fixed, based on[15]
Association between worm age and mf production rate	mf production independent of worm age	mf production independent of worm age	Assumed
Polygamy (all female worms produce mf in the presence of at least one male worm)	Yes (male potential 1000)	Yes (male potential 1000)	Assumed
Mating cycle (number of months a female can produce mf with one insemination)	1	1	Assumed

Parameter description	Anopheles model		Culex model		Source / remarks
**Uptake of infection by the vector**					
Functional relationship	$L3 = a$		$L3 = c + \frac{aM}{1 + aM/(b-c)}$		Fixed, based on [8,16]
	a	1.666	a	0.089	
	b	0.027	b	6.6	
	c	1.514	c	0	
Transmission probability (v), fraction of the L3 larvae, resulting from a single blood meal, that is released by a mosquito	0.1		0.1		Fixed, as in[8]
**Other**					
Start year of simulation period (burn-in period runs from this start-year to the moment of first intervention)	1850		1850		N/A
**Surveillance**					
Timing of surveys	Yearly, at the start of a calendaryear from 2005-2030 onwards (preceding mass treatment, when it occurs in the same year)		Yearly, at the start of a calendaryear from 2005-2030 onwards (preceding mass treatment, when it occurs in the same year)		N/A
Volume of blood examined for mf	60 µL		60 µL		N/A
Variability in observed number of mf in one 20 µl blood smear	Negative binomial distribution with k=0.33		Negative binomial distribution with k=0.33		Previously estimated for 20 µL blood by fitting to data[2]
Variation between worms in their contribution to measured mf count (dispersal factor)	Constant (no variation)		Constant (no variation)		Assumed

Parameter description	Anopheles model	Culex model	Source / remarks
**Morbidity**			
not applicable			
no excess mortality due to disease			

Table S3 – LYMFASIM assumptions related to interventions scenarios: MDA and bednet use

Parameter	Values				Source
**Mass treatment**					
Timing of mass treatment rounds, and coverage and fraction excluded per round	*Year*	*Month*	*Coverage*	*Fraction Excluded*	N/A
	2014	*0*[a]	*65%*	*5%*	
	2015	*0*[a]	*65%*	*5%*	
	2016	*0*[a]	*65%*	*5%*	
	2017	*0*[a]	*65%*	*5%*	
	2018	*0*[a]	*65%*	*5%*	
Relative compliance by age and sex					
age-group	Males		Females		Based on unpublished OCP data
0-4[b]	0[b]		0[b]		
5-9	0.75		0.5		
10-14	0.8		0.7		
15-19	0.8		0.74		
20-29	0.7		0.65		
30-49	0.75		0.7		
50+	0.8		0.75		
**Drug treatment**					
Fraction malabsorption (no effect)	0%				
Efficacy ivermectin + albendazole					
Proportion of adult worms killed per treatment, average	35%				
Duration of temporary reduction in female reproductive capacity, average	9 months[c]				

[a] Month 0 equals January 1st.

[b] Children under 5 are assumed to be exempted from treatment

Parameter		Values		Source
	Permanent reduction in female worm reproductive capacity, average	0%		
	Variability in effect of treatment on adult worms	Not applicable (assumed constant, 1)		
	Fraction of mf surviving per treatment	1%		
Efficacy diethylcarbamazine + albendazole				
	Proportion of adult worms killed per treatment, average	55%		
	Duration of temporary reduction in female reproductive capacity, average	N/A		
	Permanent reduction in female worm reproductive capacity, average	0%		
	Variability in effect of treatment on adult worms	Not applicable (assumed constant, 1)		
	Fraction of mf surviving per treatment	5%		
**Bednet use**				
	Effectivity	97% reduction in number of bites among bednet users		N/A
	Coverage by year	Year and month: moment of change in bednet coverage	Proportion of population using bednets	N/A
		1850	0	
		2006	0.16	
		2007	0.29	
		2008	0.28	
		2009	0.22	

[c] Assuming complete absence of mf production during this period and immediate resumption of mf production thereafter

Parameter	Values		Source
	2010	0.26	
	2011	0.28	
	2012	0.27	
	2013	0.30	
	2014	0.32	
	2015	0.34	
	2030	0.34	

References

1 Plaisier AP, Subramanian S, Das PK (1998). The LYMFASIM simulation program for modeling lymphatic filariasis and its control. Methods Inf Med 37: 97–108.

2 Subramanian S, Stolk WA, Ramaiah KD (2004)The dynamics of *Wuchereria bancrofti* infection: a model-based analysis of longitudinal data from Pondicherry, India. Parasitology 128: 467–482.

3 Coffeng LE, Bakker R, Montresor A, de Vlas SJ (2015) Feasibility of controlling hookworm infection through preventive chemotherapy: a simulation study using the individual-based WORMSIM modelling framework. Parasit Vectors 8: 541.

4 Stolk WA, Walker M, Coffeng LE, Basáñez M-G, de Vlas SJ. Required duration of mass ivermectin treatment for onchocerciasis elimination in Africa: a comparative modelling analysis. *Parasit Vectors* 2015; **8**: 552.

5 Stolk WA, Swaminathan S, Oortmarssen GJ van, Das PK, Habbema JDF (2003) Prospects for elimination of bancroftian filariasis by mass drug treatment in Pondicherry, India: a simulation study. J Infect Dis 188: 1371–1381.

6 Stolk WA, de Vlas SJ, Habbema JDF (2005) Anti-Wolbachia treatment for lymphatic filariasis. Lancet Infect Dis 365: 2067–2068.

7 Stolk WA, De Vlas SJ, Habbema JDF. Advances and challenges in predicting the impact of lymphatic filariasis elimination programmes by mathematical modelling. *Filaria J* 2006; **5**: 5.

8 Stolk WA, De Vlas SJ, Borsboom GJ, Habbema JDF (2008) LYMFASIM, a simulation model for predicting the impact of lymphatic filariasis control: quantification for African villages. Parasitology 135: 1583–1598.

9 Stolk WA, ten Bosch QA, de Vlas SJ, Fischer PU, Weil GJ (2013) Modeling the impact and costs of semiannual mass drug administration for accelerated elimination of lymphatic filariasis. PLoS Negl Trop Dis **7**: e1984.

10 Stolk WA, Stone C, de Vlas SJ (2015) Modelling lymphatic filariasis transmission and control: modelling frameworks, lessons learned and future directions. Adv Parasito*l* 87: 249–291.

11 Jambulingam P, Subramanian S, de Vlas SJ, Vinubala C, Stolk WA (2016) Mathematical modelling of lymphatic filariasis elimination programmes in India: required duration of mass drug administration and post-treatment level of infection indicators. Parasit Vectors 9: 501.

12 Irvine MA, Stolk WA, Smith ME (2017) Effectiveness of a triple-drug regimen for global elimination of lymphatic filariasis: a modelling study. Lancet Infect Dis 17: 451–458.

13 Smith ME, Singh BK, Irvine MA (2017) Predicting lymphatic filariasis transmission and elimination dynamics using a multi-model ensemble framework. Epidemics 18: 16–28.

14 World Health Organization (1992) Lymphatic filariasis: the disease and its control. Fifth report of the WHO Expert Committee on Filariasis. World Health Organ Tech Rep Se*r*. 821: 1–71.

15 Plaisier AP, Cao WC, Van Oortmarssen GJ, Habbema JDF. Efficacy of ivermectin in the treatment of *Wuchereria bancrofti* infection: a model-based analysis of trial results. Parasitology 119: 385–394.

16 Subramanian S, Krishnamoorthy K, Ramaiah KD, Habbema JDF, Das PK, Plaisier AP (1998) The relationship between microfilarial load in the human host and uptake and development of *Wuchereria bancrofti* microfilariae by *Culex quinquefasciatus*: a study under natural conditions. Parasitology 116: 243–255.

TRANSFIL model description and methods

Description of the mathematical model

The mathematical model of lymphatic filariasis (LF) transmission TRANSFIL is a stochastic individual-based model of LF infection in human populations. A full model description is given in Irvine *et al.*[1] and more recently in Michael *et al.*[2], so here we provide a brief summary of the model development. TRANSFIL is a stochastic individual-based model, simulating worm burden, microfilaraemia and other demographic parameters relating to age and risk of exposure. Humans are modelled individually, with their own male and female worm burden. The concentration of mf in the peripheral blood is modelled for each individual and increases according to the number of fertile female worms as well as decreasing at constant rate.

The total mf density in the population contributes towards the current density of L3 larvae in the human-biting mosquito population, where the distribution of L3 amongst the human-biting mosquito population is completely homogeneous. An empirically derived relationship is used for the uptake of mf by a mosquito, where both *Culex* and *Anopheles* uptake curves are implemented depending on setting (see Irvine *et al.*[1]), the relationship with *Anopheles* was used in the main manuscript. The model dynamics are therefore divided into the individual human dynamics, including age and turnover; worm dynamics inside the host; microfilariae dynamics inside the host and larvae dynamics inside the mosquito.

To generate the required range of mf prevalences (5 to 15% in the scenario considered), we varied two parameters of the model, the vector to host ratio (V /H) and the average population bite risk (k), using parameter sets from a range of plausible values based on previously analysed data[1,2,3]. The graphical representation of the values for the simulations that are in the 5 to 15% prevalence range (in individuals above 5 years of age) and reach the 1% mf prevalence threshold is shown in Figure S5.

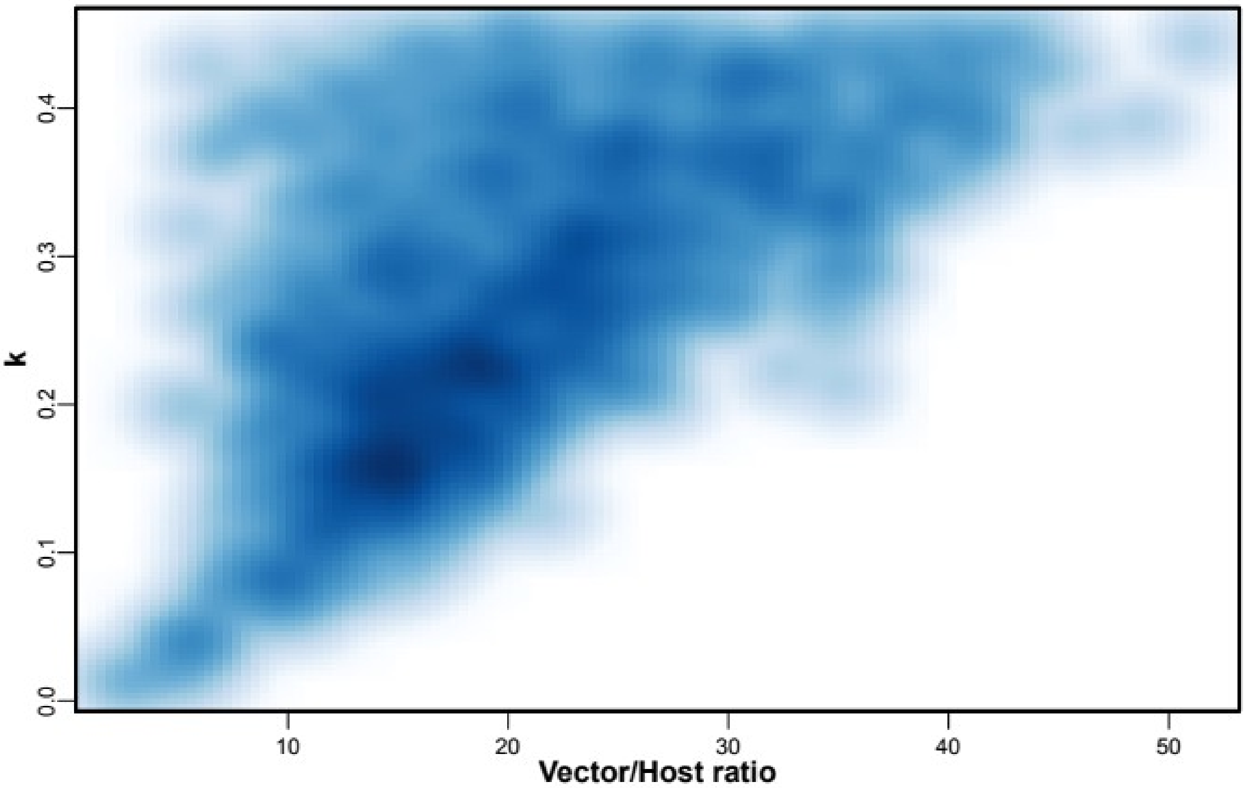

Figure S5 – Vector to Host ratio against aggregation parameter k. The density plot indicates the parameter space areas from the simulations that were in the 5 to 15% mf prevalence range at baseline and reached the 1% mf threshold after 5 rounds of MDA. Dark blue areas denote more common parameter values.

For stochastic models it is essential that an importation rate is included, otherwise the equilibrium distribution (steady state) that is used as the starting point of the simulations can potentially converge to the degenerate distribution where no-one is infected. The importation rate does not need to be large, in fact it should not be driving the infection. For this LF study we used a random number drawn from a uniform distribution with minimum 0 and maximum 0.0025 (max 2.5/1000 infections per year). The maximum importation rate here is five times smaller than previous implementation of the model[2], due to the mf prevalence range considered. The interventions reduce the prevalence over time, and therefore as year pass, the importation rate decreases based on some pilot simulations[2].

Compliance between rounds of MDA is modelled based on the paper by Griffin *et al.*[14], following the description in Dyson *et al.*[15] and previous implementation of the model[2]. A summary of all model parameters is available in table S4.

Table S4 – Description the basic LF model parameters.

Parameter symbol	Definition	Value	Source
λ	Number of bites per mosquito	10 per month	[4,5]
V/H	Ratio of number of vectors to hosts	Varied	Input
α_{max}	Age at which exposure to mosquitoes reaches its maximum level	20.0	[6]
ψ_1	Proportion of L3 leaving mosquito per bite	0.414	[7]
ψ_2	Proportion of L3 leaving mosquito that enter host	0.32	[8]
s_2	Proportion of L3 entering host that develop into adult worms	0.00275	[9,10]
μ	Death rate of adult worms	0.0104 per month	[11]
δ	Production rate of mf per worm	0.2 per month	[7]
ζ	Death rate of mf	0.1 per month	[7,12]
g	Proportion of mosquitoes which pick up infection when biting an infected host	0.37	[13]
σ	Death rate of mosquitoes	5 per month	[8]
k	Aggregation parameter of individual exposure to mosquitoes	Varied	Input
$h(\alpha)$	Parameter to adjust rate at which individuals of age α are bitten	Linear from 0 to 10, with maximum of 1	[9]

References

1. Irvine MA, Reimer LJ, Njenga SM, Gunawardena S, Kelly-Hope L, Bockarie M, and Hollingsworth TD (2015) Modelling strategies to break transmission of lymphatic filariasis -

aggregation, adherence and vector competence greatly alter elimination. Parasites and Vectors 8:547.

2. Michael E, Sharma S, Smith ME, Touloupou P, Giardina F, Prada JM, Stolk WA, Hollingsworth TD, de Vlas SJ (2018) Quantifying the value of surveillance data for improving model predictions of lymphatic filariasis elimination. PLoS Negl Trop Dis 12(10): e0006674.

3. Irvine, M.A., Stolk, W.A., Smith, M.E., Subramanian, S., Singh, B.K., Weil, G.J., Michael, E., Hollingsworth, T.D. (2017) Effectiveness of a triple-drug regimen for global elimination of lymphatic filariasis: a modelling study. Lancet Infectious Diseases 17(4), 451–458.

4. Rajagopalan P (1980) Population dynamics of culex pipiens fatigans, the filariasis vector, in pondicherry: influence of climate and environment. Proc Indian Natl Sci Acad. 6**:** 745–752.

5. Subramanian S, Manoharan A, Ramaiah K, Das P (1994) Rates of acquisition and loss of wuchereria bancrofti infection in culex quinquefasciatus. Am J Trop Med Hyg. 51: 244–249.

6. Subramanian S, Stolk W, Ramaiah K, Plaisier A, Krishnamoorthy K, Van Oortmarssen G (2004). The dynamics of wuchereria bancrofti infection: a model-based analysis of longitudinal data from Pondicherry, India. Parasitology 128: 467–482.

7. Hairston NG, de Meillon B (1968) On the inefficiency of transmission of wuchereria bancrofti from mosquito to human host. Bull World Health Organ. 38: 935.

8. Ho BC, Ewert A (1967) Experimental transmission of filarial larvae in relation to feeding behaviour of the mosquito vectors. Trans R Soc Trop Med Hyg. 61: 663–666.

9. Norman R, Chan MS, Srividya A, Pani S, Ramaiah KD, Vanamail P (2000) EPIFIL: The development of an age-structured model for describing the transmission dynamics and control of lymphatic filariasis. Epidemiol Infect. 124: 529–541.

10. Stolk WA, De Vlas SJ, Borsboom GJ, Habbema J (2008) LYMFASIM, a simulation model for predicting the impact of lymphatic filariasis control: Quantification for African villages. Parasitology 135L: 1583–1598.

11. Evans DB, Gelband H, Vlassoff C (1993) Social and economic factors and the control of lymphatic filariasis: a review. Acta Trop. 53: 1–26.

12. Ottesen E, Ramachandran C (1995) Lymphatic filariasis infection and disease: control strategies. Parasitol Today 11: 129–130.

13. Subramanian S, Krishnamoorthy K, Ramaiah K, Habbema J, Das P, Plaisier A (1998) The relationship between microfilarial load in the human host and uptake and development of

wuchereria bancrofti microfilariae by culex quinquefasciatus: a study under natural conditions. Parasitology. 116: 243–255.

14. Griffin JT, Hollingsworth TD, Okell LC, Churcher TS, White M, Hinsley W, Bousema T, Drakeley CJ, Ferguson NM, Basez MG, and Ghani AC. (2010) Reducing Plasmodium falciparum malaria transmission in Africa: a model-based evaluation of intervention strategies. PLOS Medicine. **7**: 1-17.

15. Dyson L, Stolk WA, Farrell SH, and Hollingsworth TD (2017) Measuring and modelling the effects of systematic non-adherence to mass drug administration. Epidemics 18: 56-66.

Exploring alternative scenarios

We ran the same analysis as presented in the main manuscript, but with the drug combination of diethylcarbamazine (DEC) and albendazole (ALB), DA, which is the standard regime in regions without onchocerciasis. A summary of the assumptions for the two treatment regimes is in table S5. We additionally ran simulations with *Culex* mosquitoes as the dominant vector, rather than *Anopheles* mosquitoes. The results are qualitatively similar, in regions that achieve the 1% mf threshold using DA, rather than IA, the probability of resurgence is slightly lower in the LYMFASIM model, while similar in TRANSFIL, Table S6. Results for *Culex*, rather than *Anopheles* as the dominant vector show a slightly higher resurgence probability. Trends in these settings are also qualitatively comparable, Figure S6 (a) and (b). The timing of resurgence is slower in the *Culex* simulation, compared to *Anopheles*, Figure S6 (c) and (d), which could potentially lead to missing more resurgence situations. The performance of the two metrics, mf prevalence 1 year post-MDA and the difference in mf prevalence in the time-frame between TAS-1 and TAS-2, is fairly similar, with a higher specificity (lower false positive rate) in the *Culex* simulations for the first metric, Figure S6 (e) and (f).

Table S5 – Summary of assumptions for the two treatment regimes. Alternative parametrisation of LYMFSIM shown in table S3.

Treatment Regimen	Proportion of Adult Worms Killed, %	Duration of Sterilization, mo	Proportion of Microfilariae Killed, %
DEC + ALB	55%	6	95
IVE + ALB	35%	9	99

Table S6 – Summary of resurgence probability and theoretical (for EPIFIL) and true (for LYMFASIM and TRANSFIL) elimination in the two additional settings. Confidence intervals for the resurgence probability were calculated by bootstrapping.

Result	Using DA and *Anopheles*			Using IA and *Culex*		
	EPIFIL	LYMFASIM	TRANSFIL	EPIFIL	LYMFASIM	TRANSFIL
Resurgence probability	-	0.77% (0.64-0.95%)	0.73% (0.49-0.91%)	-	1.9% (1.5-2.3%)	2.9% (2.2-3.9%)
Theoretical/ True Elimination	0	38%	16%	0	39%	23%

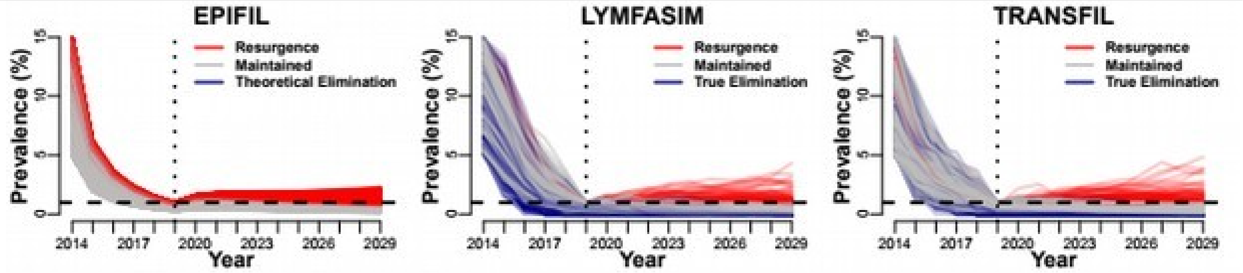

(a) DA and *Anopheles*

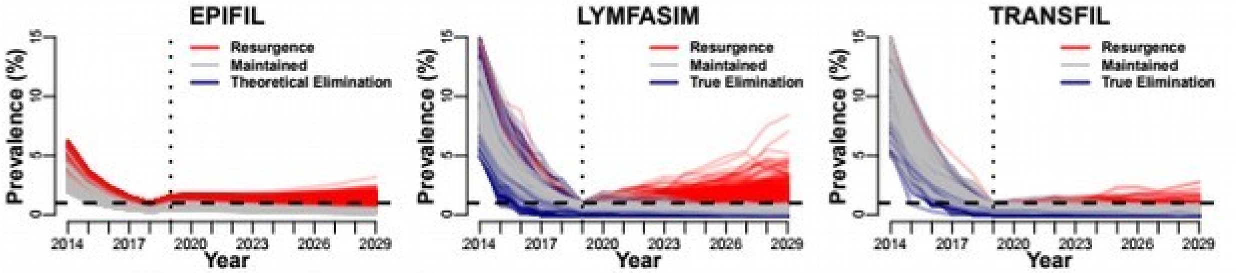

(b) IA and *Culex*

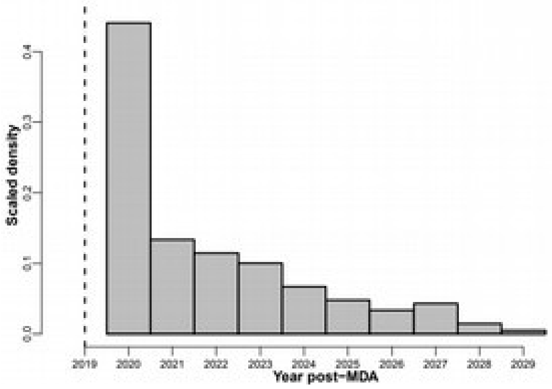

(c) DA and *Anopheles*

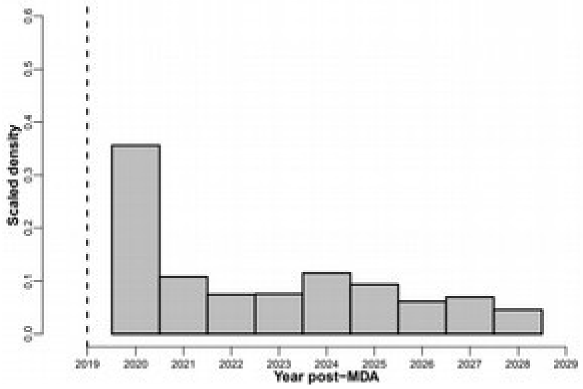

(d) IA and *Culex*

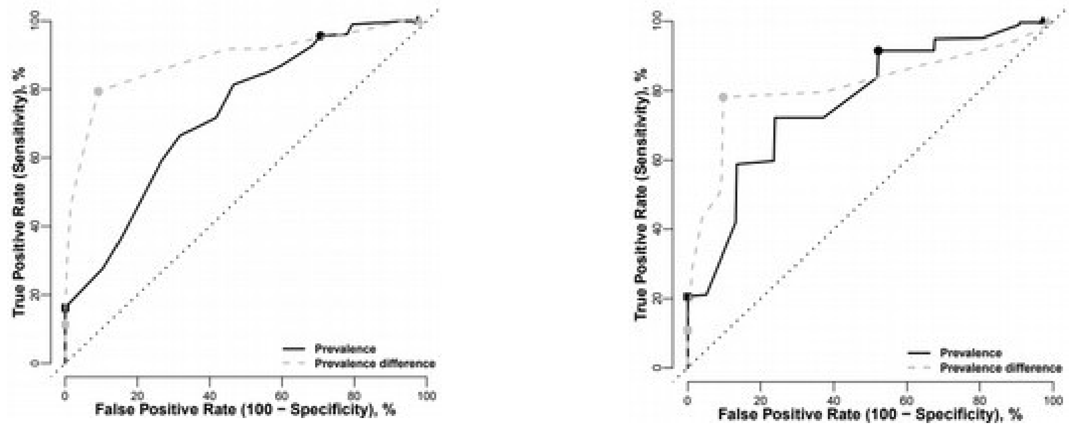

(e) DA and *Anopheles* **(f) IA and *Culex***

Figure S6 – Panel of results: (a) and (b) are temporal trends in true mf prevalence in the population for each individual run, red are the runs that are classified as resurgence, blue are the runs that achieve true elimination (0% mf prevalence); theoretical elimination in EPIFIL (0.1% mf prevalence), and in grey are runs that by 2029 remain below the threshold but have not achieved true/theoretical elimination. (c) and (d) are scaled density histogram of the year of resurgence, defined as the earliest year above the 1% mf threshold. Simulations chosen for mf prevalence of <1% one year post-MDA, in 2019, indicated by the dashed line, which represents the timing of TAS-1. (e) and (f) are receiver operating characteristic (ROC) curve showing the true positive rate against false positive rate of resurgence for a range of thresholds in two different metrics. Black line represents prevalence after MDA, with a threshold ranging between 0 and 1%. Grey line represents the difference in prevalence one year after MDA and two years later; the threshold ranges from -1% to 1% difference in prevalence. Circles show the 0.5% and zero difference threshold respectively; squares are the 1% prevalence and 0.5% prevalence difference thresholds, and triangles are the 0% and -0.5% respectively.
